# Multidrug-resistant *Neisseria gonorrhoeae* isolate, belonging to the internationally spreading Japanese FC428 clone, with ceftriaxone resistance and intermediate resistance to azithromycin, Ireland, August 2018

**DOI:** 10.2807/1560-7917.ES.2018.23.47.1800617

**Published:** 2018-11-22

**Authors:** Daniel Golparian, Lisa Rose, Almida Lynam, Aia Mohamed, Beatrice Bercot, Makoto Ohnishi, Brendan Crowley, Magnus Unemo

**Affiliations:** 1 WHO Collaborating Centre for Gonorrhoea and other Sexually Transmitted Infections, Swedish Reference Laboratory for Sexually Transmitted Infections, Department of Laboratory Medicine, Clinical Microbiology, Faculty of Medicine and Health, Örebro University, Örebro, Sweden; 2 Department of Clinical Microbiology, University of Dublin, Trinity College, St. James’s Hospital, Dublin, Ireland; 3 Guide Clinic, St. James’s Hospital, Dublin, Ireland; 4 APHP, St Louis Hospital, Laboratory of Microbiology; French National Reference Center for Bacterial STI, Associated laboratory for gonococci; Paris Diderot University, IAME, Sorbonne Paris Cité, Paris, France; 5 Department of Bacteriology I, National Institute of Infectious Diseases, Tokyo, Japan; 6 National Gonococcal Reference Laboratory, St. James’s Hospital, Dublin, Ireland; 7 Authors contributed equally to the work and share joint authorship

**Keywords:** Neisseria gonorrhoeae, gonorrhoea, treatment, ceftriaxone, azithromycin, resistance, multidrug-resistant, FC428, Ireland

## Abstract

We describe a multidrug-resistant *Neisseria gonorrhoeae* urethritis case with ceftriaxone resistance and azithromycin intermediate resistance in a heterosexual man in Ireland, August 2018. Whole-genome sequencing showed that the isolate IR72 belongs to the internationally spreading multidrug-resistant ceftriaxone-resistant FC428 clade, initially described in Japan in 2015. IR72 was assigned MSLT ST1903, NG-MAST ST17842 and NG-STAR type 1133, including the ceftriaxone resistance-mediating *penA-*60.001. Global awareness of spreading ceftriaxone-resistant gonococcal strains that threaten recommended dual therapies is essential.

We report the detailed characterisation of the first multidrug-resistant (MDR) *Neisseria gonorrhoeae* isolate with ceftriaxone resistance and intermediate resistance to azithromycin causing urethritis in a heterosexual male in Ireland in 2018. We show using whole genome sequencing (WGS) that the Irish isolate belongs to the internationally spreading MDR and ceftriaxone-resistant FC428 clone, initially described in Japan in 2015, which is further evolving [1-5].

## Case description

In August 2018, a heterosexual male presented to specialised sexually transmitted infection (STI) services in Ireland with symptoms of urethral discharge and dysuria. He reported having recent sexual contact with a female during a visit to a country in Asia. Microscopic investigation of a urethral swab revealed Gram-negative intracellular diplococci. The patient was immediately treated empirically with ceftriaxone 500 mg single intramuscular dose plus azithromycin 1 g single oral dose. A urethral swab for culture and a first-void urine sample and a pharyngeal swab for nucleic acid amplification test (NAAT; Abbott M2000 CT/NG assay) were taken. The Asian female could not be traced. The patient had no other sexual contacts since his return from Asia; and was advised to abstain sexual intercourse until follow up visit and test of cure (TOC). The culture yielded *N. gonorrhoeae* (isolate IR72) and the NAAT on the urine sample detected *N. gonorrhoeae* DNA, but the NAAT on the pharyngeal swab was *N. gonorrhoeae* negative. A TOC was performed, using NAAT on a urine sample taken 8 days after treatment, and shown to be negative 3 days later; all signs and symptoms were resolved at this follow up visit.

## Characterisation of *Neisseria gonorrhoeae* isolate IR72

Species identification of IR72 was performed using VITEK-MS (Biomérieux, Marcy l'Etoile, France) and a *porA* pseudogene PCR [6]. Antimicrobial susceptibility testing was done (in duplicate) using minimum inhibitory concentration (MIC) gradient strip tests for seven antimicrobials and results were interpreted using breakpoints stated by the European Committee on Antimicrobial Susceptibility Testing (EUCAST) [7]. The gonococcal reference strain ATCC 49226 was used for quality control; β-lactamase testing was performed as previously described [8].

IR72 showed resistance to ceftriaxone (MIC = 0.5 mg/L), cefixime (MIC = 1–2 mg/L), cefotaxime (MIC = 2–4 mg/L), ciprofloxacin (MIC > 32 mg/L), and intermediate resistance to azithromycin (MIC = 0.38–0.5 mg/L). IR72 was susceptible to spectinomycin (MIC = 16 mg/L) and tetracycline (MIC = 0.5 mg/L) and did not produce β-lactamase.

WGS was performed on Illumina MiSeq, as previously described [9]. The IR72 genome sequence was compared with previously genome-sequenced isolates from Ireland [10], and the ceftriaxone-resistant isolates FC428 from Japan [1], GK124 from Denmark [4], F90 from France [5], and the United Kingdom (UK) isolate with ceftriaxone resistance plus high-level resistance to azithromycin [11], which has been assigned as the World Health Organization (WHO) reference strain Q [NCTC 14208]. Multilocus sequence typing (MLST) (http://www.mlst.net/), *N. gonorrhoeae* multi-antigen sequence typing (NG-MAST) (http://www.ng-mast.net/), and *N. gonorrhoeae* Sequence Typing for Antimicrobial Resistance (NG-STAR) (https://ngstar.canada.ca/welcome/home) were performed using WGS data and identified sequence types (ST) ST1903, ST17842 (*porB*: 10432, *tbpB*: 21) and 1133, respectively. Regarding resistance determinants for extended-spectrum cephalosporins, IR72 harboured the extended-spectrum cephalosporins resistance-mediating mosaic *penA*-60.001 allele, which is identical to the *penA* allele in FC428, GK124, F90, and WHO Q [1,4,5,11]. Mosaic *penA*-60.001, which might originate from *N. cinerea* [12], encodes a mosaic penicillin-binding protein 2 (PBP2) including the key resistance-mediating amino acid substitutions A311V, I312M, V316T, T483S, and G545S [13]. IR72 additionally harboured the characteristic single nucleotide polymorphism (SNP; adenine) in the *mtrR* promoter inverted repeat sequence and the G120K and A121N amino acid substitutions in PorB1b, which enhance the extended-spectrum cephalosporins MICs and are associated with increased MICs of additional antimicrobials such as azithromycin, ciprofloxacin and tetracycline [13]. No 23S rRNA gene mutation associated with azithromycin resistance was found, so the intermediate azithromycin resistance was due to the *mtrR* resistance determinant and possibly additional unknown mutations. The S91F and D95A substitutions in GyrA (subunit A of DNA gyrase) and the S87R substitution in ParC (subunit C of Topoisomerase IV) caused the high-level resistance to ciprofloxacin [13]. The NG-STAR type of IR72 (1133) differs from the one of FC428 (type 233) by only one SNP in one (*porB*) of the seven NG-STAR loci. The draft genome sequence of IR72 can be found under study accession number: PRJEB29520.

The WGS phylogenomic analysis (Figure) showed that IR72 was highly different to all the previously genome-sequenced Irish isolates with decreased susceptibility or resistance to extended-spectrum cephalosporins from 2014–2016 and to WHO Q cultured in the UK in 2018 [11]. However, IR72 was belonging to the clade consisting of FC428 cultured in Japan in 2015 and the FC428 subclones identified in 2017 in Denmark (GK124 [4]) and France (F90 [5]). The whole genome of IR72 differed by 2,062 SNPs to the genome of WHO Q, but only by 60, 71, and 87 SNPs to the genomes of FC428, F90 and GK124, respectively.

**Figure:**
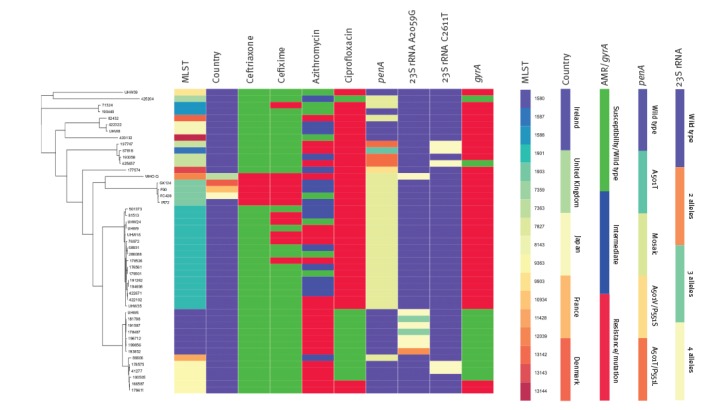
Phylogenetic tree including the whole genome sequence of the ceftriaxone-resistant isolate (IR72), Ireland, August 2018

MSLT: Multilocus sequence typing.

Phylogenetic tree based on the whole genome sequences of the ceftriaxone-resistant isolate from Ireland (IR72) and previously genome sequenced isolates with decreased susceptibility or resistance to extended-spectrum cephalosporins in Ireland [10], as well as the ceftriaxone-resistant isolates FC428 from Japan [1], GK124 from Denmark [4], F90 from France [5] and WHO Q from the UK [11]. MLST sequence types (STs), antimicrobial susceptibility to ceftriaxone, cefixime, azithromycin and ciprofloxacin, and relevant antimicrobial resistance determinants, are also shown.

The colour coding is indicated in the columns on the right.

## Discussion and conclusions

Here, we describe the detailed characterisation of the first MDR isolate with ceftriaxone resistance and intermediate resistance to azithromycin cultured from a heterosexual male with urethritis in August 2018 in Ireland. Ceftriaxone resistance in *N. gonorrhoeae* remains rare internationally [14-16]. Phenotypic and WGS characterisation showed that the Irish isolate described here belongs to the ceftriaxone-resistant and MDR FC428 clone initially described in Japan in 2015 [1]. Minor genomic changes were identified, which likely represent the evolution of FC428.

FC428 subclones were reported in 2017 in Australia, Canada, Denmark, and France [2-5] and are the first evidence of a ceftriaxone-resistant gonococcal clone that appears to have maintained a high fitness and spread internationally. Detailed examination of the phenotypic and genetic characteristics, including the fitness of the FC428 clone and its evolving subclones, is therefore imperative. Furthermore, enhanced surveillance of gonococcal antimicrobial resistance and gonorrhoea treatment failures is needed, particularly in the South-East Asian and Western Pacific Region, where FC428 and many of the FC428-associated descendants [1-5] have originated from. Most worryingly, no sexual partner(s) of the index patient in the present paper and in previous instances of infections with FC428 or its subclones [1-5] could be traced in Asia. Notably, the first gonococcal strain with ceftriaxone resistance plus high-level azithromycin resistance (WHO Q) was identified in England in 2018 [11], followed by two similar cases in Australia [17]. Two of these three cases were also associated with travel to South-East Asia [11,17].

Awareness of the international spread of FC428, its subclones and additional ceftriaxone-resistant strains that are threatening recommended dual therapies (ceftriaxone plus azithromycin) needs to be enhanced. In addition, surveillance of antimicrobial resistance and treatment failures (ideally supplemented by WGS), improved implementation of dual antimicrobial therapies with high dose of ceftriaxone and azithromycin [18], successful notification and treatment of sexual partners and TOC are essential on an international level. Further, new antimicrobials for treatment of gonorrhoea and ideally an effective gonococcal vaccine, as a long-term solution for management and control of gonorrhoea, are essential.
